# Discovery of High-Affinity Peptide Ligands Targeting the SPIN1 Tudor 3 Domain Via a Minimalist Pruning Strategy

**DOI:** 10.1021/acsmedchemlett.6c00384

**Published:** 2026-08-07

**Authors:** Alessandra Feoli, Giulio Esposito, Manuela Grimaldi, Monica Viviano, Ida Pacilio, Josef Krátký, Anna Maria D’Ursi, Ciro Milite, Haitao Li, Mark T. Bedford, Sabrina Castellano, Gianluca Sbardella

**Affiliations:** ‡Department of Pharmacy and ∫PhD Program in Drug Discovery and Development, University of Salerno, via Giovanni Paolo II 132, I-84084 Fisciano, SA, Italy; § Department of Pharmacology and Toxicology, Faculty of Pharmacy, 37740 Charles University, Akademika Heyrovského 1203, 500 05 Hradec Králové, Czech Republic; ¥ State Key Laboratory of Molecular Oncology, MOE Key Laboratory of Protein Sciences, Beijing Frontier Research Center for Biological Structure, School of Basic Medical Sciences, Tsinghua Medicine, 12442 Tsinghua University, Beijing, 100084, China; † SXMU-TM Collaborative Innovation Center for Frontier Medicine, Shanxi Medical University, Taiyuan, Shanxi Province 030001, China; # Tsinghua-Peking Center for Life Sciences, Beijing 100084, China; Δ Department of Epigenetics and Molecular Carcinogenesis, The University of Texas MD Anderson Cancer Center, Houston, Texas 77030, United States

**Keywords:** Spindlin1, SPIN1, SPINDOC, Tudor domain, peptide ligands, protein conformational changes, epigenetics

## Abstract

Spindlin1 (SPIN1) is an epigenetic reader involved in oncology, whose third Tudor domain remains largely underexplored. Here, we applied a minimalist pruning strategy to the 26-mer peptide DOCpep3 to identify the core pharmacophore for SPIN1 Tudor 3 domain binding. This yielded truncated linear peptide analogues (e.g., **1**, **3**, and **4**) displaying low nanomolar affinities, significantly outperforming the parent peptide. Orthogonal biophysical validation (MST and SPR) confirmed competitive target engagement. Furthermore, circular dichroism spectroscopy revealed that these pruned ligands induce distinct structural rearrangements in SPIN1, establishing high-affinity chemical probes for drug discovery.

## Graphical abstract

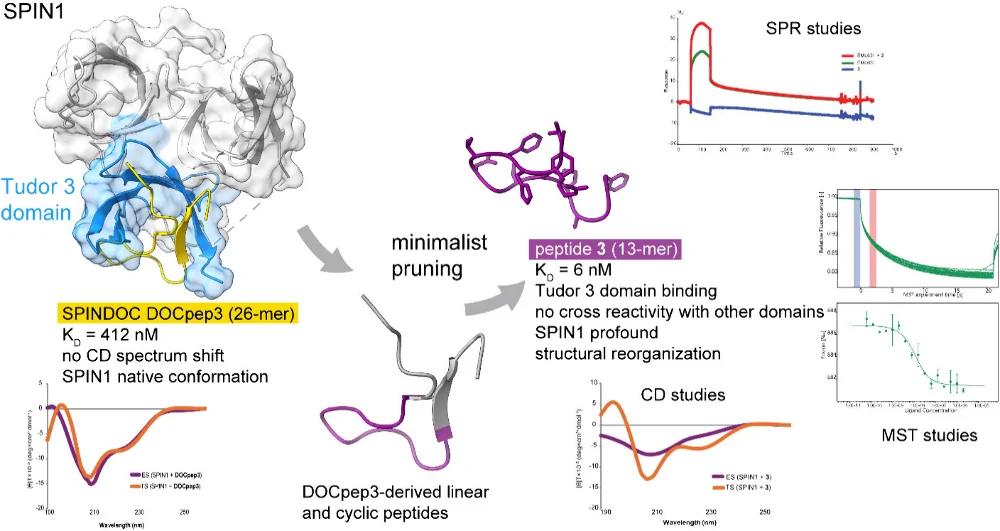

Epigenetic mechanisms play a fundamental role in modulating gene transcription, primarily through post-translational modifications (PTMs) of histones. This regulatory landscape is governed by three distinct classes of proteins: ″writers,″ which deposit specific chemical marks; ″erasers,″ which catalyze their removal; and ″readers,″ which selectively bind and interpret these modifications to influence chromatin states. Among the readers, Spindlin1 (SPIN1) is a member of the SPIN/SSTY family and a histone methylation reader involved in the regulation of gametogenesis.^1^ SPIN1 is an abundant maternal transcript in mouse oocytes and embryos at early developmental stages.^2^ Moreover, SPIN1 ^3^ regulates downstream gene transcription and is also involved in chromosome segregation during mitotic or meiotic cell division.^4,5^

Structurally, this 262-amino acid protein comprises three distinct Tudor domains, a family of motifs that mediate protein–protein interactions essential for various DNA-templated biological processes. Substrate recognition relies on an electron-rich “aromatic cage” consisting of three to five amino acids (Figure 1).^6^

**Figure 1:**
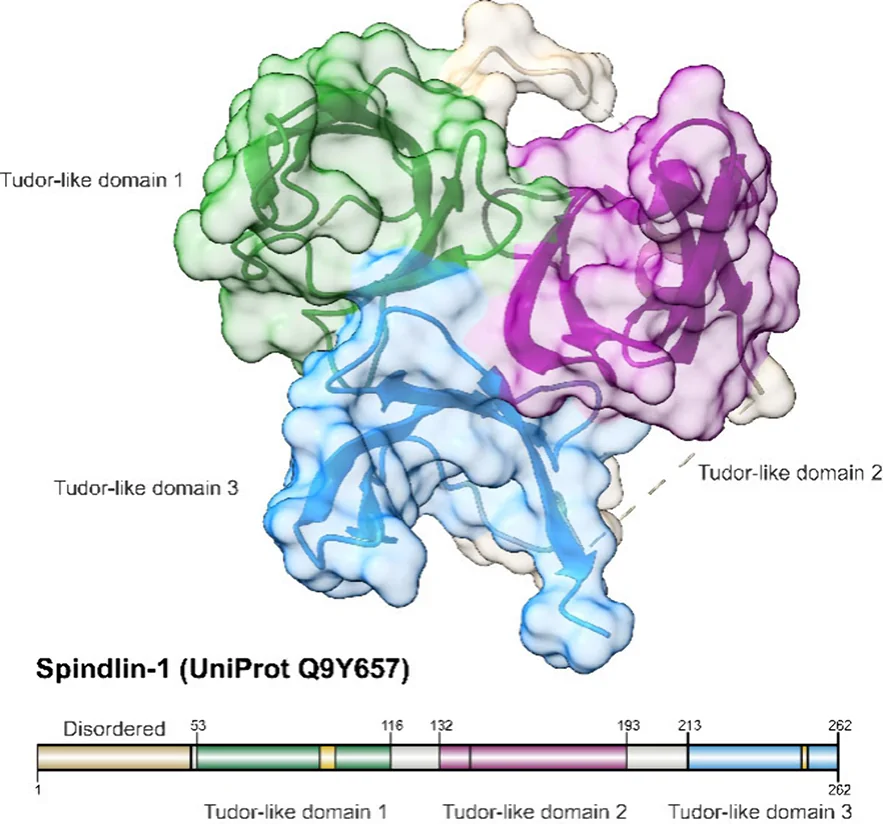
Architecture of SPIN1, showing the three Tudor domains colored in forest green, dark magenta, and dodger blue, respectively. Picture prepared using UCSF ChimeraX^7^ and Illustrator for Biological Sequences, IBS^8^ from Protein Data bank (PDB) ID code 7E9M and UniProt entry code Q9Y657 modified with Adobe Illustrator CC 2025.

Each Tudor domain in SPIN1 is characterized by a canonical β-barrel conformation of four to five antiparallel β-strands.^3^ Functionally, the first and second Tudor domains selectively recognize specific histone methylation marks.

The first Tudor domain (aromatic cage: Trp62, Trp72, Tyr91, Tyr98, Phe251)^9^ binds asymmetrically dimethylated arginine residues (Rme2a),^10^ while the second Tudor domain (aromatic cage: Phe141, Trp151, Tyr170, Tyr177)^9^ recognizes trimethylated lysine marks (K4me3, K20me3 and H3K4me3–R8me2a).^10–12^ Through the recognition of these methyl marks, SPIN1 promotes gene expression of rDNA, Wnt/TCF4 target genes, and MAZ (Myc-associated zinc finger protein)-regulated genes,^13–15^ playing a pivotal role in DNA replication, cell cycle progression, and the balance between gene activation and silencing within H3K9me3/2-enriched chromatin regions.^12,14,16–18^ The third domain lacks the critical residues to form an intact aromatic cage and cannot accommodate methyllysine binding.^18^ However, this domain interacts with distinct binding partners, such as the Hepatitis B virus protein HBx and the SPIN1 docking protein (SPINDOC), which attenuates the transcriptional coactivator role of SPIN1.^19,20^ Pathologically, SPIN1 is significantly upregulated in various cancers, including nonsmall cell lung cancer (NSCLC), breast cancer, and ovarian cancer, suggesting a function as a tumor promoter.^21^ In NSCLC, high SPIN1 expression correlates with aggressive behavior and poor patient prognosis.^22^ Moreover, SPIN1 overexpression is associated with radio and drug resistance, driving disease progression.^22–24^ SPIN1 is also linked to carcinogenesis-related cellular properties such as motility and migration; downregulating its expression or inhibiting its activity significantly impairs cancer cell cycle progression, cell proliferation, and metastasis.^25^

Functional studies confirm that SPIN1 loss suppresses cell proliferation and invasion *in vitro* and *in vivo*, and renders NSCLC cells and xenograft tumors more susceptible to irradiation by impairing DNA repair.^22^ In breast cancer, aberrant SPIN1 accumulation is frequently driven by the loss of tumor-suppressive microRNAs, including miR-148/152, miR-489, and miR-1271.^26,27^ This dysregulation is critically linked to enhanced tumor aggressiveness, maintenance of cancer stem cell self-renewal in triple-negative breast cancer (TNBC),^28^ and the acquisition of profound chemoresistance via the upregulation of drug-efflux transporters.^29^ The involvement of SPIN1 in ovarian cancer is extensively documented; originally cloned due to its marked overexpression in ovarian tumor tissues, it was historically designated as an Ovarian Cancer-Related protein (OCR).^30,31^ Mechanistically, SPIN1 is a nuclear protein that dynamically interacts with the mitotic spindle. Although physiologically required during early embryonic development, aberrant overexpression in the adult ovarian epithelium disrupts spindle organization, inducing chromosomal instability—a primary driver of malignant transformation.^30^ Furthermore, SPIN1 contributes to tumor progression by exerting a potent antiapoptotic effect. By interacting with ribosomal proteins, such as uL18, SPIN1 sequesters these complexes, preventing the activation of the uL18-MDM2-p53 signaling axis.^32^ The subsequent inhibition of p53 provides ovarian cancer cells with resistance to apoptosis, promoting survival and uncontrolled proliferation.

## Collectively, These Findings Highlight SPIN1 as a Compelling Therapeutic Target

To date, only a limited number of small-molecule inhibitors targeting SPIN1 have been reported. Our research group identified EML631 in 2017 as the first selective, cell-active SPIN1 inhibitor, exhibiting a K_D_ in the low micromolar range (4 μM).^33^ Since then, only a few compounds with higher potency have been identified (e.g., MS31,^34^ VinSpinIn,^35^ MS8535 ^36^). The high structural conservation of the histone-binding pocket presents a significant challenge in the development of SPIN1 chemical probes, as current inhibitors primarily target the aromatic cage within the first and/or second Tudor domain.

Conversely, known ligands for the SPIN1 Tudor 3 domain are limited to proteins or the 26-mer peptide derived from SPINDOC, DOCpep3 (Figure 2).^37^ While DOCpep3 exhibits a high affinity for the Tudor 3 domain, its large molecular weight and significant polar surface area limit its utility as a cellular chemical probe. Consequently, identifying the minimal structural feature required for DOCpep3 binding to SPIN1 is a critical step toward developing optimized Tudor 3 inhibitors. To delineate the minimal pharmacophore, we designed a library of seven peptide analogues (**1–7**, Figure 2) to systematically dissect the DOCpep3 interface. These rationally designed derivatives were synthesized and evaluated using biophysical techniques to assess their potency against SPIN1. Herein, we report the design, synthesis and biophysical characterization of new peptide ligands that target the SPIN1 Tudor 3 domain with low nanomolar affinity.

**Figure 2:**
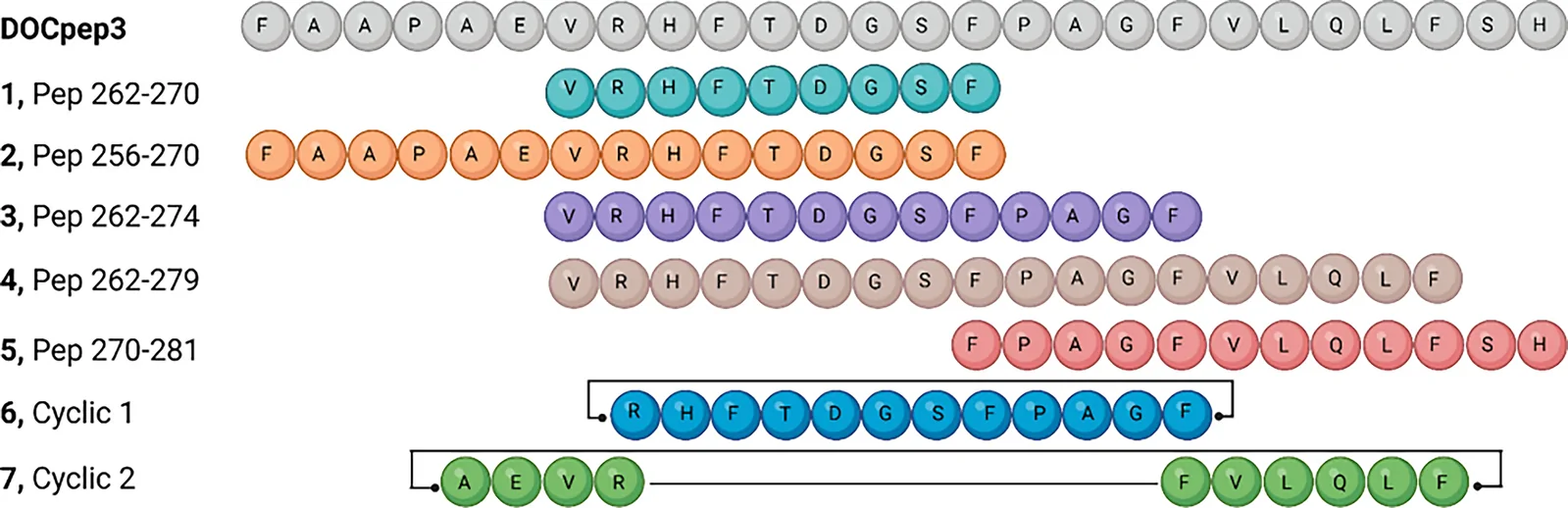
Linear (1–5) and cyclic (6,7) peptides designed from the pruning of DOCpep3. Created in BioRender. BioRender (2026) https://BioRender.com

Mechanistically, DOCpep3 engages the Tudor 3 domain via a large, discontinuous interface driven by a combination of hydrophobic anchoring and polar contacts. Specifically, peptide residues V262, F265, F270, and L276 occupy the central hydrophobic core, whereas F256, F274, and F279 serve as structural anchors at the three corners of the Tudor 3 domain. Additionally, the complex is stabilized by a network of side-chain hydrogen bonds formed between the T266-D267-S269 peptide segment and the K216-Q217 region of SPIN1 (Figure 3).

**Figure 3:**
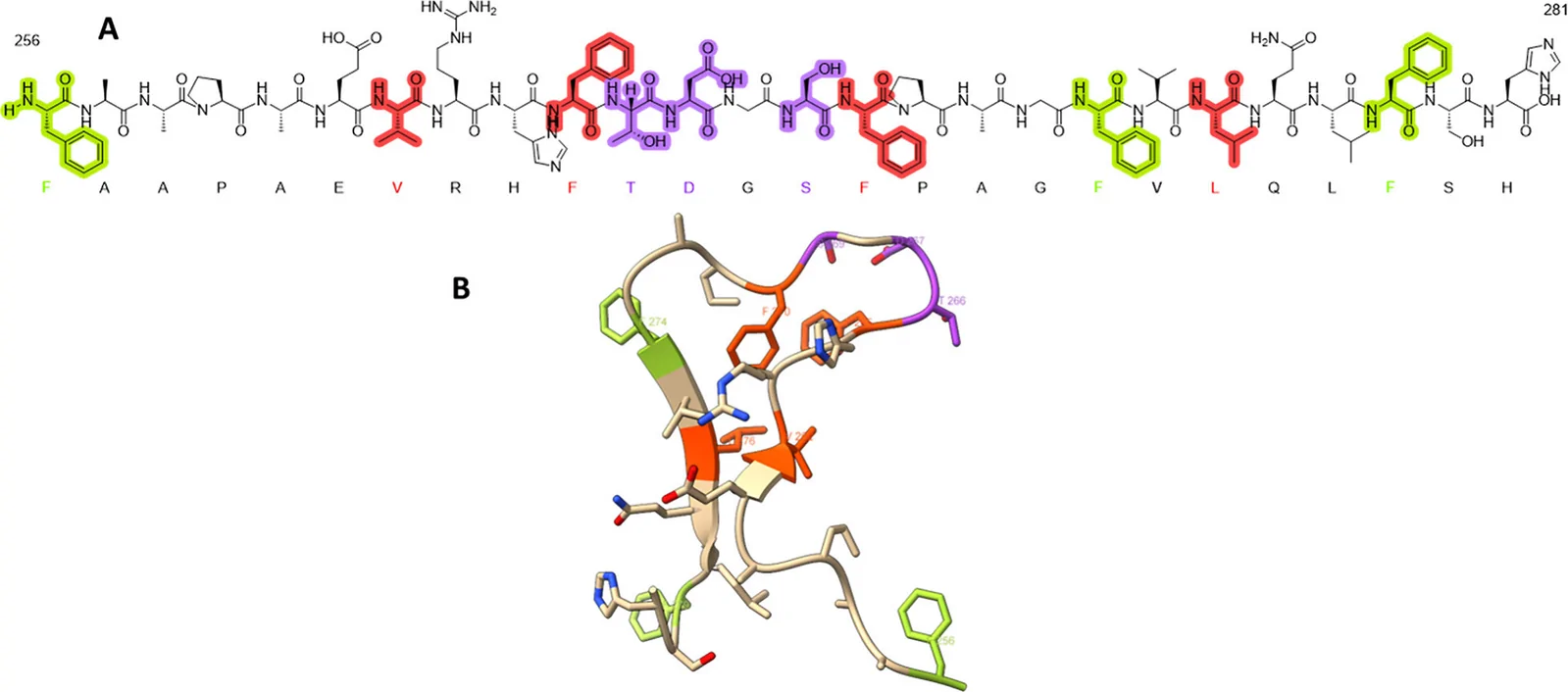
Molecular recognition of DOCpep3 by the SPIN1 Tudor 3 domain. (A) Structure of DOCpep3 with critical residues highlighted by function: red (central core engagement: V262, F265, F270, L276), green (corner anchors: F256, F274, F279), and purple (hydrogen bonding: T266, D267, S269). (B) Three-dimensional view of DOCpep3 docked within the SPIN1 binding pocket.

Based on these structural insights, we designed and synthesized a focused library of peptides to elucidate the minimal pharmacophoric requirements for targeting the SPIN1 Tudor 3 domain via systematic truncation and conformational constraints. To determine if deep pocket occupancy and polar contacts are sufficient for binding in the absence of the peripheral phenylalanine anchor regions, we designed peptide **1** (262–270). This short peptide isolates the central hydrophobic core and hydrogen-bonding region while eliminating all three phenylalanine anchors.

Subsequently, to evaluate the individual contributions of the phenylalanine anchoring network, we prepared a series of N- and C-terminally elongated variants (peptides **2–4**) that systematically reintroduce these anchor residues. Specifically, peptide **2** (256–270) incorporates the N-terminus up to the first phenylalanine (F256) residue. Peptide **3** (262–274) partially restores the C-terminus of DOCpep3 to include the central phenylalanine (F274), testing whether stabilizing this central loop is sufficient to sustain molecular recognition. Peptide **4** (262–279) represents a near-complete reconstruction of the C-terminus of DOCpep3, incorporating the central core alongside both the central (F274) and distal (F279) phenylalanines.

In contrast, peptide **5** (270–281) was designed to evaluate whether the isolated C-terminal hydrophobic region could constitute an independent binding motif.

Finally, to overcome the inherent conformational flexibility and limitations of linear peptides, we developed two cyclic analogues. Peptide **6** structurally constrains the key hydrophobic and polar residues (263–274) into a macrocyclic scaffold, whereas peptide **7** bridges the N-terminal (260–263) and C-terminal (274–279) hydrophobic elements together. Peptides **1**-**7** were initially screened for their ability to bind recombinant human SPIN1 (aa 50–262), using Microscale Thermophoresis (MST). For these evaluations, SPIN1 was covalently labeled on lysine residues and maintained at a fixed concentration (10 nM), while the peptide analytes were evaluated across a range of concentrations. DOCpep3 and the small-molecule inhibitor EML631 were employed as reference positive controls. The calculated dissociation constants K_D_ are summarized in Table 1, and the corresponding MST binding curves are provided in Figure S1 (Supporting Information).

**Table 1:** MST Binding Experiments Results

Peptides	K_D_ (μM)^a^
**EML631**	0.479 ± 0.245
**DOCpep3 (256–281)**	0.412 ± 0.019
**1 (262–270)**	0.011 ± 0.002
**2 (256–270)**	0.192 ± 0.024
**3 (262–274)**	0.006 ± 0.001
**4 (262–279)**	0.042 ± 0.019
**5 (270–281)**	n.b.^b^
6 (Cyclic 1–12)	15.9 ± 5.9
7 (Cyclic 1–10)	n.b.^b^

a SPIN1 was fluorescently labeled on lysines using RED-NHS second generation dye (NanoTemper). Peptides were serially diluted in PBS-T and mixed 1:1 with 20 nM SPIN1. Each mixture was incubated at room temperature for 15 min before measurement. MST experiments were analyzed using high MST power and a LED intensity of 20%. K_D_ values were calculated from compound concentration-dependent changes in normalized fluorescence (F_norm_).

b n.b.= no binding

Remarkably, the linear truncated peptides **1**-**4**, which retain the central hydrophobic core and hydrogen-bonding motif, displayed significantly higher affinity than the full-length reference compound DOCpep3. This validates our pruning strategy as a highly effective approach for optimizing ligands against the SPIN1 protein.

Specifically, analogues **1**, **3**, and **4** emerged as the most potent ligands, exhibiting low nanomolar K_D_ values (K_D_ = 0.011 ± 0.002 μM, K_D_ = 0.006 ± 0.001 μM and K_D_ = 0.042 ± 0.019 μM, respectively). Peptide **2**, which reintroduces the amino-terminal region of DOCpep3, showed a lower affinity for SPIN1 (K_D_ = 0.192 ± 0.024 μM) compared to **1**, **3**, and **4**. This reduction in potency suggests that the N-terminal sequence imposes unfavorable constraints that shift its binding profile closer to that of the parent DOCpep3. Conversely, peptide **5** failed to show any binding, demonstrating that the isolated C-terminal hydrophobic region is incapable of independently engaging SPIN1.

Regarding the macrocyclic variants, peptide **6** exhibited a severely compromised affinity (15.9 ± 5.9 μM), indicating that this specific conformational constraint is poorly tolerated, while peptide **7** showed no detectable binding to SPIN1.

To mitigate potential artifacts and the intrinsic technical limitations associated with individual biophysical methods, the validation of target engagement via orthogonal assays is critical in drug discovery pipelines.

Therefore, we employed Surface Plasmon Resonance (SPR) as an orthogonal method to validate our MST findings. For the SPR assays, SPIN1 was covalently immobilized onto a CM5 sensor chip via standard amine coupling. The synthetic peptides were flowed over the chip surface at varying concentrations, and steady-state affinity analysis was applied to determine equilibrium K_D_ values. Consistent with the MST protocols, DOCpep3 and EML631 served as positive controls.

As reported in Table 2, all tested peptides demonstrated binding to SPIN1 in the SPR assay, with affinities spanning the low- to midmicromolar range.

**Table 2:** SPR Binding Experiments Results

Peptides	K_D_ (μM)^a^
**EML631**	12.95 ± 7.57
**DOCpep3 (256–281)**	9.27 ± 0.26
**1 (262–270)**	0.25 ± 0.07
**2 (256–270)**	3.30 ± 0.19
**3 (262–274)**	0.12 ± 0.02
**4 (262–279)**	0.11 ± 0.04
**5 (270–281)**	0.30 ± 0.02
**6 (Cyclic1–12)**	3.50 ± 2.26
**7 (Cyclic1–10)**	22.55 ± 3.61

a SPIN1 was immobilized on a CM5 sensor chip by amine-coupling reaction. Compounds, diluted in PBS-T buffer, were injected at different concentrations with an association and a dissociation time of 90 and 200 s, respectively, and with a flow rate of 30 μL/min. PBS-T was used as running buffer. Sensorgrams obtained for each compound were double referenced, subtracting blank injections and the reference flow cell. For the determination of the equilibrium K_D_, a steady state affinity analysis was applied.

In agreement with the MST data, linear peptides **1**–**4** outperformed the parent macro-peptide DOCpep3 in SPR, corroborating the critical importance of the central hydrophobic and hydrogen bonding networks for driving target engagement. Moreover, peptides **1**, **3**, and **4** were confirmed as the top-performing candidates within the series, further demonstrating that truncation of the N-terminal region optimizes the binding interaction with SPIN1. Interestingly, peptide **5**, which was classified as a nonbinder in MST, unexpectedly demonstrated an affinity comparable to peptide **1** in the SPR assay (K_D_ = 0.30 ± 0.02 μM). This discrepancy underscores the necessity of utilizing multiple cross-validation biophysical platforms, as relying on a single technique can yield misleading false negatives. Furthermore, the SPR results for cyclic analogue **6** (K_D_ = 3.50 ± 2.26 μM) verified our previous findings that this cyclization approach is a nonviable design strategy, while peptide **7** was confirmed as the weakest binder in the series (K_D_ = 22.55 ± 3.61 μM).

When comparing absolute affinities, the K_D_ values derived from MST were consistently lower than those obtained via SPR. This variance is unsurprising given that MST and SPR rely on entirely different physical principles, experimental setups, and surface immobilization states (solution-phase vs matrix-tethered). Crucially, however, both methods produced highly consistent trends and ranking orders across the library.

Analysis of the SPR sensorgrams (Figure SI2, Supporting Information) revealed that while DOCpep3 and peptide **7** exhibited typical dose-dependent positive response curves, peptides **1**–**6** generated atypical negative sensorgrams. Standard ligand–receptor binding models dictate that an increase in analyte concentration over an immobilized protein surface should produce a proportional increase in positive response units (RU). However, anomalous negative sensorgrams have been documented in the literature, particularly when analytes interact with targets immobilized within three-dimensional polymeric dextran matrices, such as CM4 or CM5 sensor chips. Such negative signals reflect specific, authentic receptor–ligand interactions that are accompanied by pronounced conformational rearrangements in the protein matrix upon analyte binding.^38–40^

As detailed by Bonnet et al.,^41^ these nonconventional negative signals originate from a negative deviation in the refractive index increment (RII) of the ligand-analyte complex, which counterbalances and overpowers the mass-dependent signal increase expected from target recognition. Because thermodynamic equilibrium data can still be accurately extracted from these curves, steady-state affinity analysis was applied to compute the K_D_ values.

Taken together, the negative profiles observed for **1**–**6** strongly suggest that these pruned peptides engage SPIN1 via a distinct binding mode that triggers significant conformational shifts in the protein, contrasting with the behavior of DOCpep3 and **7**.

To definitively confirm these binding-induced conformational changes, we performed Circular Dichroism (CD) spectroscopy. Analogous to our SPR observations, peptide **3** was selected as a representative candidate from the negative-sensorgram subset, while DOCpep3 was used as a control. CD spectra were recorded for individual components (Figure 4A and 4C) and their corresponding mixtures (Figure 4B and 4D). Conformational variations were analyzed by comparing the experimental spectrum of the protein-peptide mixture against a calculated theoretical sum spectrum (derived by adding the isolated spectra of the individual components).^42^ For the DOCpep3–SPIN1 system, the theoretical and experimental curves overlaid tightly without major deviations (Figures 4B). Conversely, a striking divergence was observed between the theoretical and experimental curves for the peptide **3**–SPIN1 mixture (Figures 4D).

**Figure 4:**
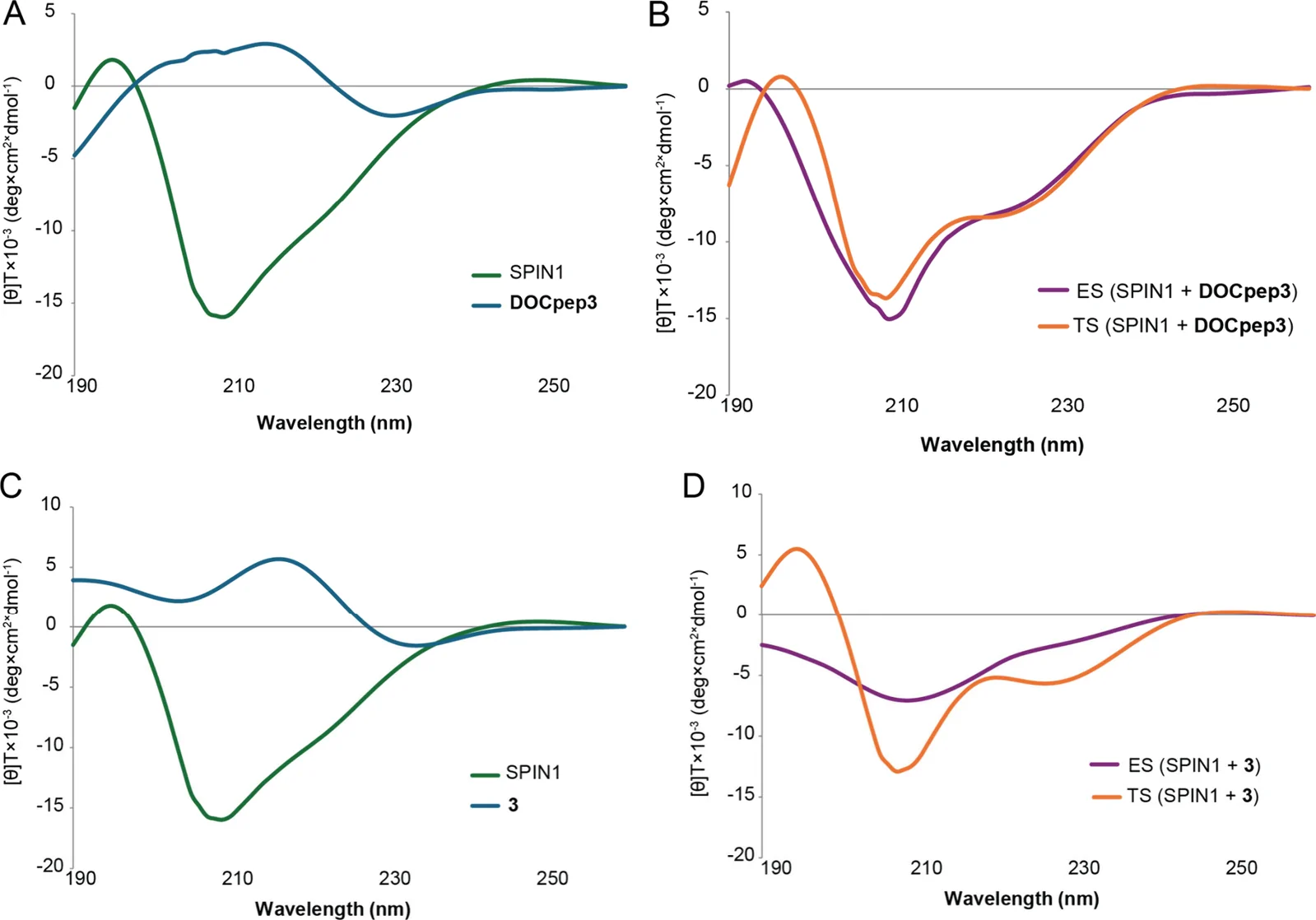
Circular Dichroism experiments. A: spectrum of SPIN1 and DOCpep3. B: theoretical spectrum (TS) and experimental spectrum (ES) of DOCpep3-SPIN1 mixtures. C: spectrum of SPIN1 and 3. D: theoretical spectrum (TS) and experimental spectrum (ES) of 3-SPIN1 mixtures.

Dichroweb deconvolution of the CD data indicated that native SPIN1 consists of 75% α-helix, 14% turns, and 11% unordered structures. Incubation with DOCpep3 left these secondary structure fractions essentially unchanged, showing only a nominal 1% decrease in α-helix content in favor of β-strand alignment. In sharp contrast, the addition of peptide **3** triggered a profound structural reorganization of SPIN1, shifting the secondary structure composition to 31% α-helix, 8% β-strand, 36% turns, and 25% unordered conformations. These spectroscopic findings provide robust biophysical proof that peptide **3** induces major conformational adjustments in SPIN1 upon binding, validating the mechanism hypothesized from our SPR sensorgram deviations.

Although prior crystallographic studies confirmed that DOCpep3 engages the third Tudor domain of SPIN1,^37^ it was necessary to verify if our truncated variants target the identical binding pocket. Thus, we conducted SPR-based competition assays using peptide **3** and DOCpep3.

By coinjecting one peptide at a saturating concentration alongside the second peptide at a concentration equivalent to 2 × K_D_, competitive vs noncompetitive profiles could be distinguished. If both ligands compete for the same binding cavity, the combined mixture should generate a signal response identical to that of the saturating peptide alone. If they occupy distinct, nonoverlapping pockets, the mixture will yield an additive response higher than the saturating compound alone.

Experimentally, sequential injections of a saturating concentration of DOCpep3, peptide **3** (2 × K_D_), and their mixture were evaluated. The green trace of the mixture matched the red trace of DOCpep3 alone, clearly demonstrating a purely competitive binding mechanism (Figure 5A). As a control experiment, we evaluated the competition between DOCpep3 and EML631—a small molecule known to target the first and second Tudor domains of SPIN1, but not Tudor 3.^33^

**Figure 5:**
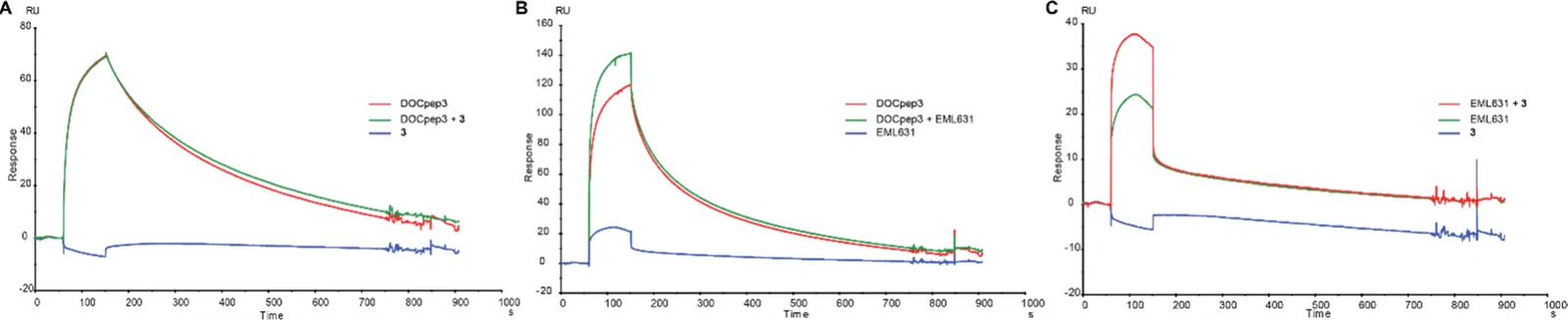
A) Competition experiment between DOCpep3 and peptide 3 by SPR. DOCpep3 was injected at a saturating concentration while peptide 3 at a concentration 2× its K_D_. B) Competition experiment between DOCpep3 and EML631 by SPR. DOCpep3 was injected at a saturating concentration while EML631 at a concentration 2× its K_D_. C) Competition experiment between peptide 3 and EML631 by SPR. EML631 was injected at a saturating concentration while peptide 3 at a concentration 2× its K_D_.

As expected, the mixture generated an elevated, additive signal profile indicative of noncompetitive binding (Figure 5B). Similarly, cross-competition assays between peptide **3** and EML631 confirmed a noncompetitive relationship (Figure 5C). These competitive profiles correlate with established structural and crystallographic data, validating this SPR methodology as a reliable tool for mapping binding site topology. Collectively, these findings demonstrate that despite its dramatically reduced molecular footprint, peptide **3** selectively and competitively targets the identical Tudor 3 pocket as the parent DOCpep3 macro-peptide.

## Conclusion

SPIN1 represents a highly compelling therapeutic target for oncology. Nevertheless, this reader protein remains significantly underexplored, and the few small-molecule inhibitors reported to date target the highly conserved aromatic cages of the first and second Tudor domains exclusively.

In this study, we exploited the known binding mode of DOCpep3 to design and prepare a compact library of peptide analogues utilizing systematic truncation and conformational constraints. This structural pruning strategy successfully mapped the minimal pharmacophoric core required to target the SPIN1 Tudor 3 domain.

Orthogonal biophysical validation via MST and SPR demonstrated that linear fragments retaining the central hydrophobic core and hydrogen-bonding motifs—most notably peptides **1**, **3**, and **4**—display superior binding affinities relative to the parent DOCpep3 macro-peptide.

Interestingly, SPR and CD analyses revealed that these pruned, low-molecular-weight peptides trigger an unprecedented conformational rearrangement in SPIN1 upon binding. Furthermore, SPR competition assays verified that peptide **3** competitively shares the Tudor 3 pocket with DOCpep3, showing no cross-reactivity with binders targeting alternative domains, such as EML631.

In summary, this minimalist pruning strategy has yielded downsized, high-affinity peptide ligands with nanomolar potencies, establishing a crucial milestone toward the development of optimized, drug-like chemical probes for the SPIN1 Tudor 3 domain.

No unexpected or unusually high safety hazards were encountered.

## Supplementary Material



## Notes

Experimental procedures for peptide synthesis, purification, and characterization, SPIN1 expression, MST and SPR binding experiments, SPR competition experiments, CD experiments (PDF)

## Abbreviations

CD: Circular Dichroism
IBS: Illustrator for Biological Sequences
KD: equilibrium dissociation constant
MAZ: Myc-associated zinc finger protein
MST: Microscale Thermophoresis
NSCLC: nonsmall cell lung cancer
OCR: Ovarian Cancer-Related protein
PDB: Protein Data bank
PTMs: post-translational modifications
RII: refractive index increment
Rme2a: asymmetrically demethylated arginine residues
RU: response units
SPIN1: Spindlin1
SPINDOC: SPIN1 docking protein
SPR: Surface Plasmon Resonance
TNBC: triple-negative breast cancer
uL18: universal large ribosomal subunit protein 18

## References

[1] Staub E., Mennerich D., Rosenthal A. (2001). The Spin/Ssty repeat: a new motif
identified in proteins involved in vertebrate development from gamete
to embryo. Genome Biol..

[2] Oh B., Hwang S. Y., Solter D., Knowles B. B. (1997). Spindlin, a major
maternal transcript expressed in the mouse during the transition from
oocyte to embryo. Development.

[3] Zhao Q., Qin L., Jiang F., Wu B., Yue W., Xu F., Rong Z., Yuan H., Xie X., Gao Y. (2007). Structure of human spindlin1. Tandem tudor-like domains for cell
cycle regulation. J. Biol. Chem..

[4] Choi J. W., Zhao M. H., Liang S., Guo J., Lin Z. L., Li Y. H., Jo Y. J., Kim N. H., Cui X. S. (2017). Spindlin
1 is essential for metaphase II stage maintenance and chromosomal
stability in porcine oocytes. Mol. Hum. Reprod..

[5] Zhang P., Cong B., Yuan H., Chen L., Lv Y., Bai C., Nan X., Shi S., Yue W., Pei X. (2008). Overexpression
of spindlin1 induces metaphase arrest and chromosomal instability. J. Cell Physiol.

[6] Cipriano A., Sbardella G., Ciulli A. (2020). Targeting epigenetic reader domains
by chemical biology. Curr. Opin. Chem. Biol..

[7] Meng E. C., Goddard T. D., Pettersen E. F., Couch G. S., Pearson Z. J., Morris J. H., Ferrin T. E. (2023). UCSF ChimeraX:
Tools for structure
building and analysis. Protein Sci..

[8] Liu W., Xie Y., Ma J., Luo X., Nie P., Zuo Z., Lahrmann U., Zhao Q., Zheng Y., Zhao Y. (2015). IBS: an illustrator
for the presentation and visualization of biological
sequences. Bioinformatics.

[9] Vorreiter C., Robaa D., Sippl W. (2024). Exploring Aromatic
Cage Flexibility
Using Cosolvent Molecular Dynamics Simulations–An In-Silico
Case Study of Tudor Domains. J. Chem. Inf. Model..

[10] Su X., Zhu G., Ding X., Lee S. Y., Dou Y., Zhu B., Wu W., Li H. (2014). Molecular basis underlying histone H3 lysine-arginine
methylation pattern readout by Spin/Ssty repeats of Spindlin1. Genes Dev..

[11] Wang C., Zhan L., Wu M., Ma R., Yao J., Xiong Y., Pan Y., Guan S., Zhang X., Zang J. (2018). Spindlin-1 recognizes methylations of K20 and R23 of histone H4 tail. FEBS Lett..

[12] Wang W., Chen Z., Mao Z., Zhang H., Ding X., Chen S., Zhang X., Xu R., Zhu B. (2011). Nucleolar
protein Spindlin1 recognizes H3K4 methylation and stimulates the expression
of rRNA genes. EMBO Rep.

[13] Franz H., Greschik H., Willmann D., Ozretić L., Jilg C. A., Wardelmann E., Jung M., Buettner R., Schüle R. (2015). The histone
code reader SPIN1 controls RET signaling
in liposarcoma. Oncotarget.

[14] Wang J. X., Zeng Q., Chen L., Du J. C., Yan X. L., Yuan H. F., Zhai C., Zhou J. N., Jia Y. L., Yue W. (2012). SPINDLIN1 promotes cancer
cell proliferation through
activation of WNT/TCF-4 signaling. Mol. Cancer
Res..

[15] Zhang X., Zhu G., Su X., Li H., Wu W. (2018). Nucleolar localization
signal and histone methylation reader function is required for SPIN1
to promote rRNA gene expression. Biochem. Biophys.
Res. Commun..

[16] Wang C., Zhan L., Wu M., Ma R., Yao J., Xiong Y., Pan Y., Guan S., Zhang X., Zang J. (2018). Spindlin-1 recognizes methylations of K20 and R23 of histone H4 tail. FEBS Lett..

[17] Zhao F., Liu Y., Su X., Lee J.-E., Song Y., Wang D., Ge K., Gao J., Zhang M. Q., Li H. (2020). Molecular basis for
histone H3 “K4me3-K9me3/2” methylation pattern readout
by Spindlin1. J. Biol. Chem..

[18] Yang N., Wang W., Wang Y., Wang M., Zhao Q., Rao Z., Zhu B., Xu R. M. (2012). Distinct mode of methylated lysine-4
of histone H3 recognition by tandem tudor-like domains of Spindlin1. Proc. Natl. Acad. Sci. U. S. A..

[19] Du Y., Yan Y., Xie S., Huang H., Wang X., Ng R. K., Zhou M. M., Qian C. (2021). Structural mechanism of bivalent
histone H3K4me3K9me3 recognition by the Spindlin1/C11orf84 complex
in rRNA transcription activation. Nat. Commun..

[20] Liu W., Yao Q., Su X., Deng Y., Yang M., Peng B., Zhao F., Du C., Zhang X., Zhu J. (2023). Molecular insights into
Spindlin1-HBx interplay and its impact on
HBV transcription from cccDNA minichromosome. Nat. Commun..

[21] Li D., Guo J., Jia R. (2021). Histone code reader SPIN1 is a promising target of
cancer therapy. Biochimie.

[22] Zhong M., Fang Z., Zou J., Chen X., Qiu Z., Zhou L., Le Y., Chen Z., Liao Y., Nie F. (2024). SPIN1
accelerates tumorigenesis and confers radioresistance
in non-small cell lung cancer by orchestrating the FOXO3a/FOXM1 axis. Cell Death Dis.

[23] Yang Z., Wu H., Zhang K., Rao S., Qi S., Liu M., Chen Y., Wang Y. (2022). Circ_0007580 knockdown
strengthens
the radiosensitivity of non-small cell lung cancer via the miR-598-dependent
regulation of THBS2. Thorac Cancer.

[24] Wang Y., Li M., Chen Y., Jiang Y., Zhang Z., Yan Z., Liu X., Wu C. (2024). SPIN1 facilitates chemoresistance and HR repair by
promoting Tip60 binding to H3K9me3. EMBO Rep.

[25] Song Q., Ji Q., Xiao J., Li F., Wang L., Chen Y., Xu Y., Jiao S. (2018). miR-409 Inhibits Human Non-Small-Cell Lung Cancer Progression
by Directly Targeting SPIN1. Mol. Ther Nucleic
Acids.

[26] Chen X., Wang Y. W., Xing A. Y., Xiang S., Shi D. B., Liu L., Li Y. X., Gao P. (2016). Suppression of SPIN1-mediated PI3K-Akt
pathway by miR-489 increases chemosensitivity in breast cancer. J. Pathol.

[27] Du H. Y., Liu B. (2018). MiR-1271 as a tumor suppressor in breast cancer proliferation and
progression via targeting SPIN1. Eur. Rev. Med.
Pharmacol Sci..

[28] Drago-Ferrante R., Pentimalli F., Carlisi D., De Blasio A., Saliba C., Baldacchino S., Degaetano J., Debono J., Caruana-Dingli G., Grech G. (2017). Suppressive
role exerted by microRNA-29b-1–5p in triple negative breast
cancer through SPIN1 regulation. Oncotarget.

[29] Chen X., Wang Y. W., Gao P. (2018). SPIN1, negatively regulated
by miR-148/152,
enhances Adriamycin resistance via upregulating drug metabolizing
enzymes and transporter in breast cancer. J.
Exp Clin Cancer Res..

[30] Gao Y., Yue W., Zhang P., Li L., Xie X., Yuan H., Chen L., Liu D., Yan F., Pei X. (2005). Spindlin1,
a novel nuclear protein with a role in the transformation of NIH3T3
cells. Biochem. Biophys. Res. Commun..

[31] Jiang F., Zhao Q., Qin L., Pang H., Pei X., Rao Z. (2006). Expression, purification, crystallization and preliminary X-ray analysis
of human spindlin1, an ovarian cancer-related protein. Protein Pept Lett..

[32] Fang, Z. ; Cao, B. ; Liao, J. M. ; Deng, J. ; Plummer, K. D. ; Liao, P. ; Liu, T. ; Zhang, W. ; Zhang, K. ; Li, L. ; SPIN1 promotes tumorigenesis by blocking the uL18 (universal large ribosomal subunit protein 18)-MDM2-p53 pathway in human cancer. Elife 2018, 7. DOI: 10.7554/eLife.31275. PMC5871334 29547122

[33] Bae N., Viviano M., Su X., Lv J., Cheng D., Sagum C., Castellano S., Bai X., Johnson C., Khalil M. I. (2017). Developing Spindlin1 small-molecule inhibitors
by using protein microarrays. Nat. Chem. Biol..

[34] Xiong Y., Greschik H., Johansson C., Seifert L., Bacher J., Park K. S., Babault N., Martini M., Fagan V., Li F. (2019). Discovery of a Potent and Selective Fragment-like Inhibitor
of Methyllysine Reader Protein Spindlin 1 (SPIN1). J. Med. Chem..

[35] Fagan V., Johansson C., Gileadi C., Monteiro O., Dunford J. E., Nibhani R., Philpott M., Malzahn J., Wells G., Faram R. (2019). A Chemical Probe for Tudor Domain Protein Spindlin1
to Investigate Chromatin Function. J. Med. Chem..

[36] Xiong Y., Greschik H., Johansson C., Seifert L., Gamble V., Park K. S., Fagan V., Li F., Chau I., Vedadi M. (2024). Discovery of a Potent, Selective, and Cell-Active SPIN1
Inhibitor. J. Med. Chem..

[37] Zhao F., Deng Y., Yang F., Yan Y., Feng F., Peng B., Gao J., Bedford M. T., Li H. (2024). Molecular
Basis for SPINDOC-Spindlin1 Engagement and Its Role in Transcriptional
Attenuation. J. Mol. Biol..

[38] Mesch S., Moser D., Strasser D. S., Kelm A., Cutting B., Rossato G., Vedani A., Koliwer-Brandl H., Wittwer M., Rabbani S. (2010). Low molecular
weight
antagonists of the myelin-associated glycoprotein: synthesis, docking,
and biological evaluation. J. Med. Chem..

[39] Stokmaier D., Khorev O., Cutting B., Born R., Ricklin D., Ernst T. O., Boni F., Schwingruber K., Gentner M., Wittwer M. (2009). Design, synthesis and
evaluation of monovalent ligands for the asialoglycoprotein receptor
(ASGP-R). Bioorg. Med. Chem..

[40] Crauste C., Willand N., Villemagne B., Flipo M., Willery E., Carette X., Dimala M. M., Drucbert A. S., Danze P. M., Deprez B. (2014). Unconventional
surface plasmon resonance signals reveal
quantitative inhibition of transcriptional repressor EthR by synthetic
ligands. Anal. Biochem..

[41] Bonnet H., Coche-Guerente L., Defrancq E., Spinelli N., Van der
Heyden A., Dejeu J. (2021). Negative SPR Signals during Low Molecular
Weight Analyte Recognition. Anal. Chem..

[42] Rodger A., Marrington R., Roper D., Windsor S. (2005). Circular dichroism
spectroscopy for the study of protein-ligand interactions. Methods Mol. Biol..

