# Supplementary material for: Discovery of High-Affinity Peptide Ligands Targeting the SPIN1 Tudor 3 Domain Via a Minimalist Pruning Strategy
Source: ACS Med Chem Lett. 2026 Aug 7;17(9):2066–73. doi: 10.1021/acsmedchemlett.6c00384 (PMC13573250; doi:10.1021/acsmedchemlett.6c00384)
Supplement: Supplementary file 1 [file ml6c00384_si_001.pdf]

Discovery of High-Affinity Peptide Ligands Targeting the SPIN1 Tudor 3 Domain via a Minimalist Pruning Strategy

Alessandra Feoli,[‡] Giulio Esposito,[‡,∫] Manuela Grimaldi,[‡] Monica Viviano,[‡] Ida Pacilio,[‡,∫] Josef Krátký,[‡,§] Anna Maria D'Ursi,[‡] Ciro Milite,[‡,*] Haitao Li,[¥,†,#] Mark T. Bedford,[∆] Sabrina Castellano,[‡] Gianluca Sbardella.[‡,*]

[‡]Department of Pharmacy and [∫]PhD Program in Drug Discovery and Development, University of Salerno, via Giovanni Paolo II 132, I-84084 Fisciano (SA), Italy

[§]Department of Pharmacology and Toxicology, Faculty of Pharmacy, Charles University, Akademika Heyrovského 1203, 500 05 Hradec Králové, Czech Republic

[¥]State Key Laboratory of Molecular Oncology, MOE Key Laboratory of Protein Sciences, Beijing Frontier Research Center for Biological Structure, School of Basic Medical Sciences, Tsinghua Medicine, Tsinghua University, Beijing, 100084, China

[†]SXMU-TM Collaborative Innovation Center for Frontier Medicine, Shanxi Medical University, Taiyuan, Shanxi Province 030001, China

[#]Tsinghua-Peking Center for Life Sciences, Beijing 100084, China

[∆]Department of Epigenetics and Molecular Carcinogenesis, The University of Texas MD Anderson Cancer Center, Houston, Texas 77030, United States

*C.M.: E-mail, cmilite@unisa.it; G.S.: E-mail, gsbardella@unisa.it

Table of Contents:

Materials and Methods — Page S2

Figure SI1. Thermophoretic curves for DOCpep3 and peptides **1**-**4** and **6** — Page S4

Figure SI2. Sensorgrams obtained from SPR experiments — Page S5

Materials and Methods

Peptides synthesis

All peptides were synthesized by Pepmic Co., Ltd., Suzhou, China. Briefly, linear peptides (**1-5**) were synthesized using standard 9-fluorenylmethoxycarbonyl (Fmoc) solid-phase peptide synthesis (SPPS) on a 2-chlorotrityl chloride (2-CTC) resin. Peptide chain elongation was achieved through sequential cycles of Fmoc deprotection, using 20% piperidine in *N,N*-dimethylformamide (DMF), and amino acid coupling, mediated by *N,N'*-diisopropylcarbodiimide (DIC) and 1-hydroxybenzotriazole (HOBt). Upon completion of the sequence, the peptides were cleaved from the resin and globally deprotected using a cleavage cocktail consisting of 95% trifluoroacetic acid (TFA), 1% H2O, 2% 1,2-ethanedithiol (EDT), and 2% triisopropylsilane (TIS) for 2 hours. The crude peptides were precipitated in ice-cold ether, centrifuged, and lyophilized. Purification was performed by preparative reverse-phase high-performance liquid chromatography (RP-HPLC) using a water/acetonitrile gradient containing 0.1% TFA. Final peptide purity (>95%) and molecular weights were confirmed by analytical HPLC and liquid chromatography-mass spectrometry (LC-MS), respectively

For the synthesis of the cyclic peptide **6,7**, an orthogonal protection strategy was employed during the Fmoc-SPPS process. The initial amino acid (Fmoc-Glu-OAll) was anchored to the 2-CTC resin. Following the sequential assembly of the linear peptide chain as described above, the orthogonal allyl (OAll) protecting group was selectively removed using tetrakis(triphenylphosphine)palladium(0). After the removal of the final N-terminal Fmoc group, on-resin macrocyclization was promoted by the addition of DIC and HOBt, allowing the reaction to proceed for 12 hours under a nitrogen atmosphere. The cyclized peptide was then washed, cleaved from the resin, deprotected, and precipitated using the same TFA-based cleavage cocktail described for the linear sequences. Purification of the crude cyclic peptide was performed via preparative RP-HPLC to achieve a final purity of >95%, followed by lyophilization

SPIN1 expression and purification

SPIN1 (aa 50-262) was in house expressed and purified. Plasmid pGEX-4T-1 encoding SPIN1, was transformed in BLR(DE3)pLysS cells, and the protein was expressed in lysogeny broth (LB) in the presence of 100 μg/mL ampicillin. Cells were grown at 37 °C to OD_{600} of 0.8 and induced by isopropyl-1-thio-D-galactopyranoside (final concentration, 100 μM) and incubated for 20 hours at 16 °C. Cells were harvested, pelleted at 5,000 x *g* for 15 min at 4 °C, washed twice with PBS pH 7.4, and frozen at −80°C. Before protein purification, the pellet was thawed on ice, suspended in 40 mL of lysis buffer (PBS pH 7.4 supplemented with protease inhibitors cocktail). Cells were lysed using a sonicator (Vibra-Cells, Sonics) (amplitude 30%) for 20 min on ice, and cell debris were pelleted at 10,000 x g for 30 min at 4 °C. The clarified lysate was filtered using a 0.45 μm syringe filter and loaded onto a 1 mL GSTrap HP column at the flow rate of 1 mL/min, previously conditioned with the binding buffer (PBS pH 7.4). The protein was eluted in isocratic mode using the elution buffer (50 mM Tris-HCl, 10 mM reduced glutathione, pH 8.0). Fractions containing the protein were confirmed by sodium dodecyl sulfate-polyacrylamide gel electrophoresis (SDS-PAGE), pooled, and dialyzed overnight at 4 °C to remove glutathione.

MST binding experiments

SPIN1 (aa 50-262) was labeled following the protocol for N-hydroxysuccinimide (NHS) coupling of (NanoTemper) to lysine residues of the protein. Briefly, 90 μL of a 10 μM solution of SPIN1 protein was mixed with 10 μL of 300 μM second generation NT-647 fluorophore, both diluted in labeling buffer (130 mM $NaHCO_3$, 50 mM NaCl, pH 8.2), and incubated for 30 min at room temperature in the dark. Unbound fluorophores were removed by size-exclusion chromatography using as running buffer PBS. The concentration of the protein after labeling was determined using extinction coefficient $\varepsilon = 82927.5\ M^{-1}\ cm^{-1}$ for SPIN1, with a correction factor C_{corr} of 0.04 at 280 nm. After the labeling procedure, we obtained 1.37 μM protein and a degree of labelling (DOL) of 1. For storage, labeled SPIN1 was frozen in 20 μL aliquots at –80 °C. Prior to MST experiments, aliquots of labeled SPIN1 were thawed on ice and centrifuged at 12000 × *g* for 15 min (at 4 °C) to remove protein aggregates. Pretests using standard-treated capillaries (NanoTemper)

were performed to test for adsorption of protein to capillary walls by analyzing capillary scans recorded by the Monolith NT.115pico (NanoTemper), prior to MST experiments. The protein did not adsorb to standard-treated capillary walls in PBS buffer supplemented with 0.05 % Tween 20, hence MST experiments were performed using these capillaries. The binding of compounds to SPIN1 were performed on a Monolith NT.115pico instrument. Peptides stock in H_2O were serially diluted in PBS-T. Each dilution was mixed 1:1 with a solution of 20 nM SPIN1 to yield a final volume of 20 μL (and a final concentration of 10 nM) and incubated at room temperature for 15 minutes. Subsequently, the reaction mixtures were loaded into standard treated capillaries and subsequently analysed by high MST power and a light-emitting diode (LED) intensity of 20%. K_D values were calculated from compound concentration-dependent changes in normalized fluorescence (Fnorm) of SPIN1 after 2.5 s of thermophoresis. Data were analysed using *MO Affinity Analysis software* (NanoTemper).

SPR binding experiments

SPR experiments were performed on a Biacore T200 biosensor (Cytiva). SPIN1 (aa 50-262) protein was diluted at a concentration of 50 μg/mL in 10 mM sodium acetate, pH 4.5 and then immobilized on a CM5 sensor chip using standard amine-coupling protocols, using HBS-P as running buffer. At the end of the procedure, a density of 9000 RU was obtained. One flow cell was left empty for background subtractions. For the binding experiments, PBS buffer (phosphate-buffered saline, pH 7.4) supplemented with 0.05 % Tween-20 was used as the running buffer. Compounds were diluted in PBS supplemented with 0.05 % Tween-20. Binding experiments were performed at 25 °C using a flow rate of 30 μL/min, with 90 s monitoring of association and 200 s monitoring of dissociation. The sensorgrams obtained for each compound were double referenced, subtracting blank injections and the reference flow cell. For the determination of K_D values, the BiaEvaluation software (Cytiva) was employed, and a steady state affinity analysis was applied.

SPR competition experiments

SPR experiments were performed on a Biacore T200 biosensor (Cytiva). SPIN1 was immobilized on a CM5 sensor chip using the same procedure reported for binding experiments. For competition experiments PBS buffer (phosphate-buffered saline, pH 7.4) supplemented with 0.05 % Tween-20 was used as the running buffer. Compounds were diluted in PBS supplemented with 0.05 % Tween-20. Competition experiments between **DOCpep3** and peptide **3** were performed by sequentially injecting the solution of **DOCpep3** at 100 μM, peptide **3** at 0.24 μM, and a mixture of both. Competition experiments between **DOCpep3** and **EML631** were performed by sequentially injecting the solution of **DOCpep3** at 100 μM, **EML631** at 25 μM, and a mixture of both. Competition experiments between **EML631** and peptide **3** were performed by sequentially injecting the solution of **EML631** at 100 μM, peptide **3** at 0.24 μM, and a mixture of both. Binding experiments were performed at 25 °C using a flow rate of 30 μL/min, with 90 s monitoring of association and 600 s monitoring of dissociation. The sensorgrams obtained for each compound were corrected subtracting the reference flow cell.

CD experiments

For CD experiment we dissolved the protein SPIN1, the peptide **3** and **DOCpep3** in PBS buffer. The final concentration of the protein was 4 μM, while the concentration of peptide **3** and **DOCpep3** was 125 μM and 40 μM, respectively. **DOCpep3** was used as negative control. The CD spectra were obtained using a JASCO J-810 spectropolarimeter, with the aid of a 1 mm long quartz cell and working at a temperature of 25 °C. The CD spectra were obtained by an average of 4 scans, in a measuring range 260–190 nm, at a bandwidth of 1 nm and, at a scanning speed of 10 nm/min. Each spectrum was processed by subtracting the solvent spectrum. The processing of the CD curves was performed using Spectra Analysis tool in the *JASCO* software (JASCO Corp., Tokyo, Japan).

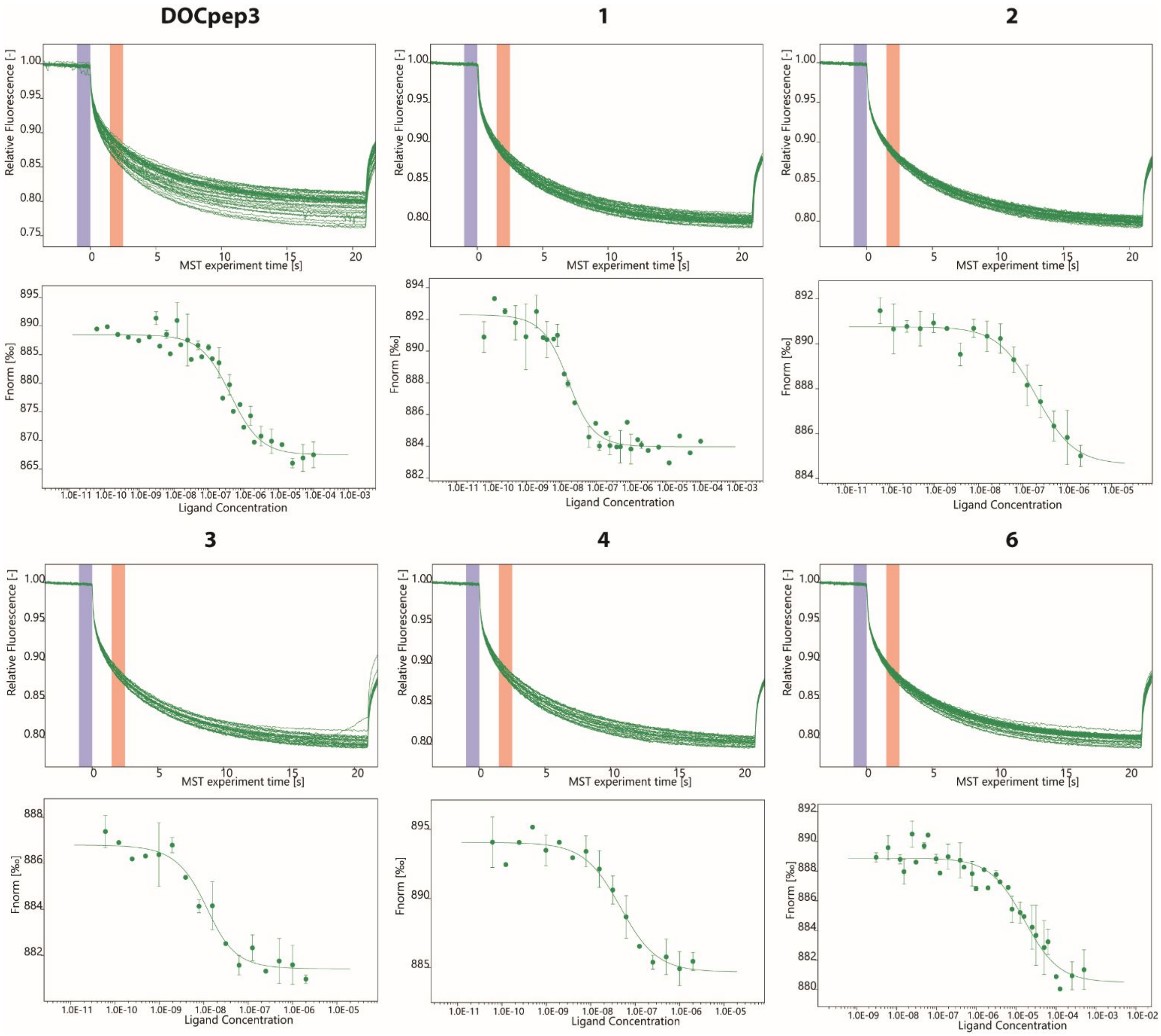

Figure SI1. Thermophoretic curves and sigmoidal dose-response fitting obtained for DOCpep3 and peptides **1-4** and **6.** For these binding experiments, SPIN1 was covalently labeled on lysines with second generation NT-647 RED dye and used at fixed concentration (10 nM), while the peptides were serially diluted. Data was analysed using MO Affinity Analysis software (NanoTemper).

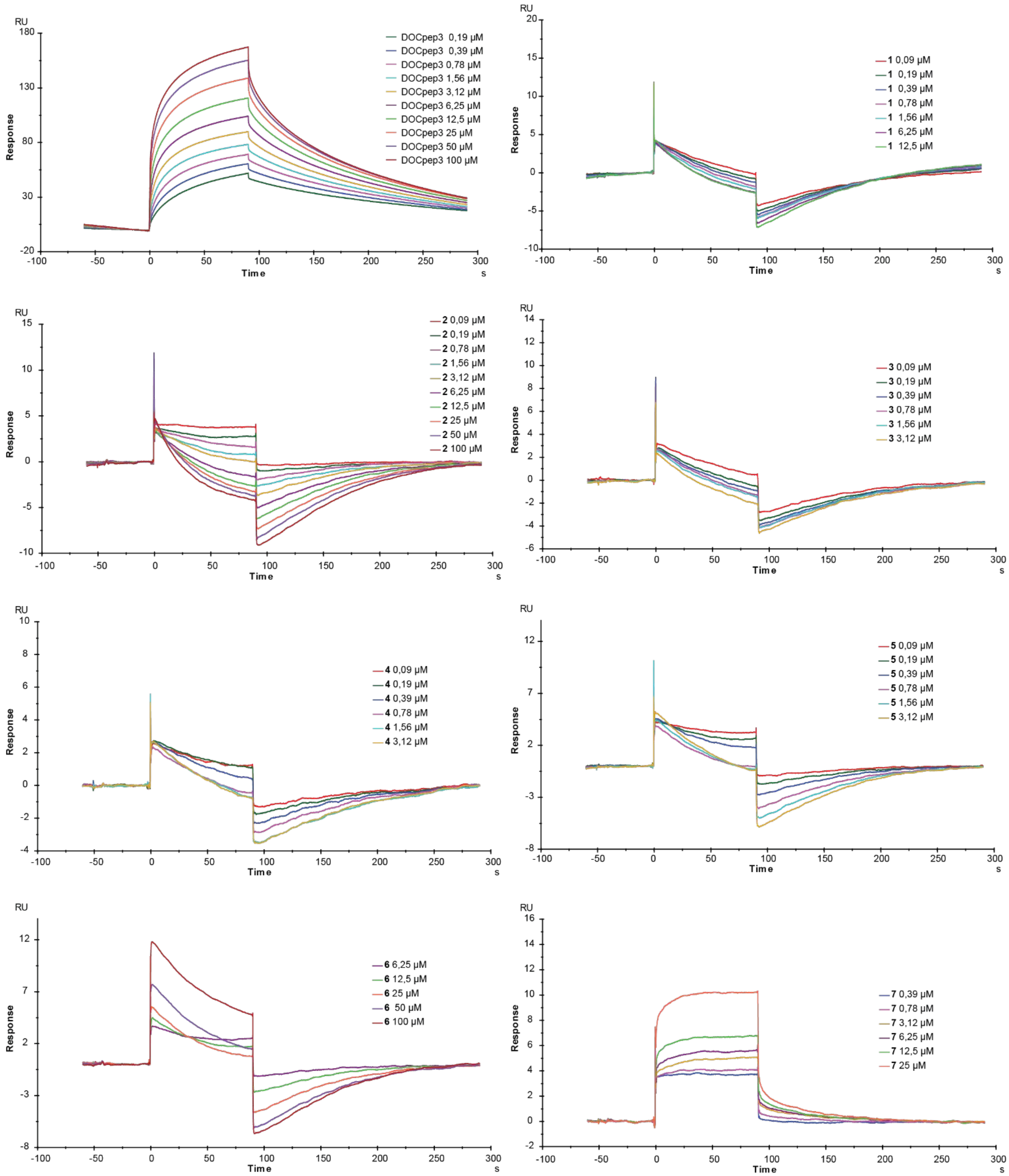

Figure SI2. Sensorgrams obtained from SPR experiments. SPIN1 was covalently attached on the sensor chip by amine coupling reaction, exploiting amine groups of lysine. Each peptide was injected at different concentrations, with a contact time of 90 seconds and a dissociation time of 200 seconds. For **DOCpep3** and peptide **7**, we obtained positive curves, while for the peptides **1-6** we observed negative sensorgrams, suggesting a putative conformational change of the protein.
